# Optogenetic stimulation of mPFC pyramidal neurons as a conditioned stimulus supports associative learning in rats

**DOI:** 10.1038/srep10065

**Published:** 2015-05-14

**Authors:** Guang-yan Wu, Guo-long Liu, Hui-min Zhang, Chong Chen, Shu-lei Liu, Hua Feng, Jian-feng Sui

**Affiliations:** 1 Department of Physiology, College of Basic Medical Sciences, Third Military Medical University, Chongqing 400038, China; 2 Experimental Center of Basic Medicine, College of Basic Medical Sciences, Third Military Medical University, Chongqing 400038, China; 3 Department of Neurosurgery, Southwest Hospital, Third Military Medical University, Chongqing 400038, China

## Abstract

It is generally accepted that the associative learning occurs when a behaviorally neutral conditioned stimulus (CS) is paired with an aversive unconditioned stimulus (US) in close temporal proximity. Eyeblink conditioning (EBC) is a simple form of associative learning for motor responses. Specific activation of a population of cells may be an effective and sufficient CS for establishing EBC. However, there has been no direct evidence to support this hypothesis. Here, we show in rats that optogenetic activation of the right caudal mPFC pyramidal neurons as a CS is sufficient to support the acquisition of delay eyeblink conditioning (DEC). Interestingly, the associative memory was not stably expressed during the initial period of daily conditioning session even after the CR acquisition reached the asymptotic level. Finally, the intensity and consistency of the CS were found to be crucial factors in regulating the retrieval of the associative memory. These results may be of importance in understanding the neural cellular mechanisms underlying associative learning and the mechanisms underlying retrieval process of memory.

Eyeblink conditioning (EBC) is a simple form of associative learning that provides a powerful model system for investigating the plasticity of neuronal circuits and the mechanisms underlying associative learning, as the basic underlying circuitry has been thoroughly studied over the last few decades^0^. The EBC involves paired presentations of a behaviorally neutral conditioned stimulus (CS; e.g., a tone or light) and an aversive unconditioned stimulus (US; e.g., a corneal airpuff or periorbital shock). According to the temporal relationship between the CS and the US, two procedures are commonly used in EBC: trace and delay paradigms. In the trace eyeblink conditioning (TEC), a temporal gap occurs between the offset of the CS and the onset of the US, which is in contrast to the delay eyeblink conditioning (DEC), in which the CS overlaps the US and the two stimuli are terminated at the same time^1^.

An important question in neuroscience is how a distinct memory is formed and stored in the brain^2^. Accumulating evidence supports the idea that specific brain regions are responsible for a specific memory. For example, while the cerebellum and the related brainstem nuclei are essential and sufficient for the simple DEC^5^, the medial prefrontal cortex (mPFC) lesions impaired EBC with non-optimal training parameters, such as during DEC with a soft tone CS^0^, suggesting the mPFC may play potential roles in DEC. However, to further understand the role of mPFC and the important cellular mechanisms underlying DEC, it is necessary to conduct a mimicry experiment to investigate whether activation of a population of cells in mPFC as a CS is sufficient for establishing DEC. However, there has been no direct evidence to support this hypothesis. Though several lines of direct evidence suggest that the intensity of CS plays an important role in the acquisition process of EBC^6^, the role of the CS intensity in retrieval process of EBC remains poorly known. Moreover, knowledge about the role of CS consistency in retrieval process of EBC remains sparse. Here, we investigated whether optogenetic activation of a subpopulation of pyramidal neurons in the right caudal mPFC as a CS paired with a periorbital shock US is sufficient to acquire DEC in rats, and evaluated the characteristics of the associative memory and the roles of CS intensity and consistency in the associative memory retrieval.

## Results

### Successful acquisition of DEC

**Figure 1:**
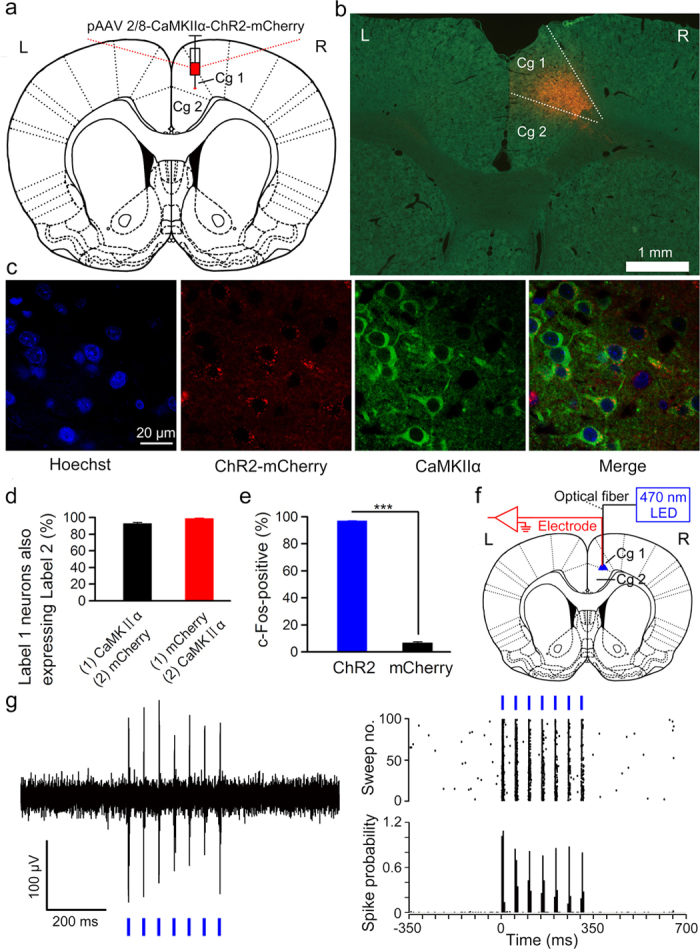
Selective labelling and optogenetic activation of the right caudal mPFC neurons. (**a**) The rats were stereotactically injected with pAAV 2/8-CaMKIIα-ChR2-mCherry targeting the right caudal mPFC. (**b**) Example of ChR2-mCherry expression in the right caudal mPFC. (**c**) Representative images showing cell-specific ChR2-mCherry expression (red) in pyramidal neurons (green) of the right caudal mPFC. (**d**) Statistics of expression in the right caudal mPFC pyramidal neurons (502 cells, from five mice). (**e**) Percentage of c-Fos-positive cells among ChR2-mCherry-expressing cells (324/334 cells) or mCherry-expressing cells (21/320 cells) after light stimulation (n = 3 rats each; ^***^P < 0.001; two-tailed unpaired Student’s t-test). (**f**) *In vivo* right caudal mPFC “optrode” recording setup. (**g**) Multi-unit activity in the right caudal mPFC from a rat injected with pAAV 2/8-CaMKIIα-ChR2-mCherry in response to trains of 7 light pulses (470 nm, 10 mW/mm^2^, 20 Hz, 10 ms pulse duration). Blue bars represent light on. Data are represented as mean ± s.e.m.

To label and activate a subpopulation of pyramidal neurons in the right caudal mPFC, we stereotactically injected rats with plasmid adeno-associated virus (pAAV) encoding both channelrhodopsin-2 (ChR2) and mCherry protein or only mCherry protein under control of the calcium/calmodulin-dependent protein kinase IIα (CaMKIIα) promoter into the right caudal mPFC (Fig. 1a,b). Following injection of the virus into the right caudal mPFC, the cell-type specificity of expression was assayed using immunocytochemistry. 92.8 ± 1% CaMKIIα-expressing pyramidal neurons expressed ChR2-mCherry (Fig. 1c,d) and the promoter provided high specificity as well, because 98.6 ± 0.4% ChR2-mCherry-expressing cells were CaMKIIα-expressing cells and no obvious overlap was detected between ChR2-mCherry-expressing neurons and GABA (γ-aminobutyric acid)-expressing neurons (Fig. 1d and Supplementary Fig. S1). In addition, almost all of the ChR2-mCherry–expressing cells in the right caudal mPFC (rats were injected with pAAV 2/8-CaMKIIα-ChR2-mCherry; the ChR2 group) were also positive for endogenous c-Fos after optical stimulation (Fig. 1e and Supplementary Fig. S2). However, only very few mCherry-expressing cells in the right caudal mPFC (rats were injected with pAAV 2/8-CaMKIIα-mCherry; the mCherry group) were c-Fos positive after optical stimulation (Fig. 1e and Supplementary Fig. S2). The proportion of c-Fos-positive cells in the right caudal mPFC was significantly greater in the ChR2-mCherry group compared with the mCherry group after optical stimulation. Next, we performed an optrode consisting of a fiber optic cannula with a multi-wire electrode (insulated nichrome wires, 17.78 μm inner diameter) tightly coupled with a 200 μm core diameter fiber to simultaneously deliver light and record neuronal activity of the right caudal mPFC in anesthetized rats 3–4 weeks after virus injection (Fig. 1f). As expected, ChR2-expressing cells showed robust responses to light stimulation (470 nm, 350 ms, 10 mW/mm^2^, 20 Hz, 10 ms pulse duration) of 8 rats in the ChR2 group (Fig. 1g). In contrast, we did not record any light-responsive cells of 7 rats in the mCherry group (data not shown).

**Figure 2:**
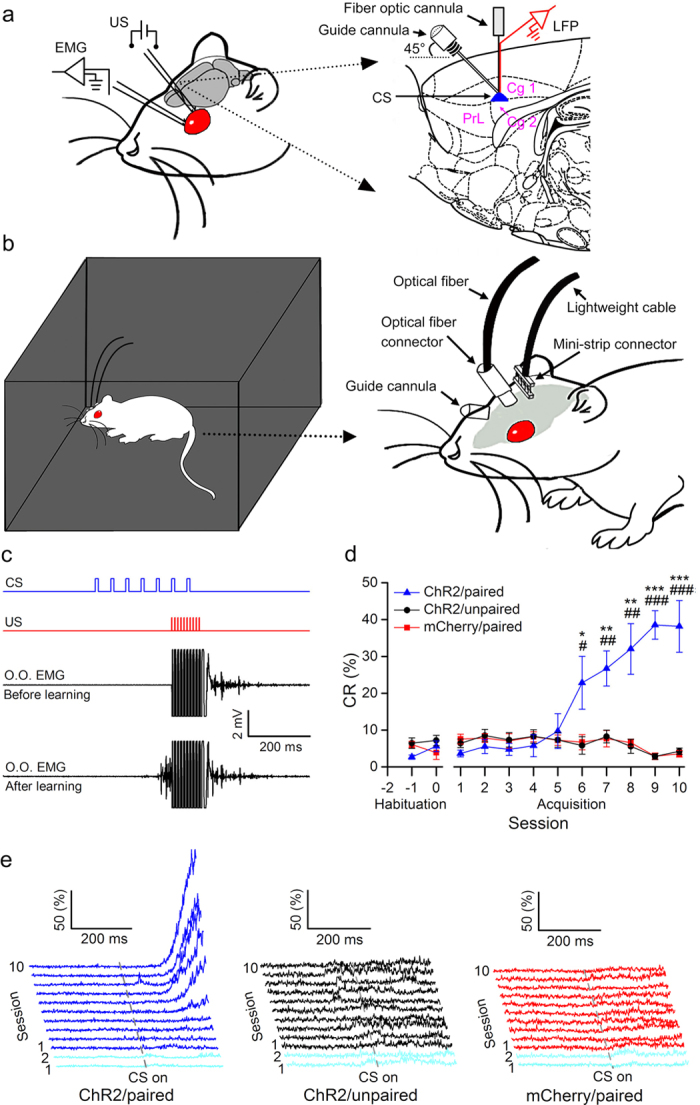
Optogenetic stimulation CS supports the acquisition of associative eyeblink conditioning. (**a**) Rats were implanted with stimulating electrodes in the subdermally caudal to the left eye for delivery of unconditioned stimuli (US) and with electrodes for recording the electromyographic (EMG) activity of the ipsilateral orbicularis oculi (O.O.) muscle. An optrode and a guide cannula were targeted to the right caudal mPFC for optical stimulation, for recording local field potentials, and for drug injection. (**b**) Rats were trained in a sound- and light-attenuating chamber. (**c**) Upper panel: the conditioning paradigm illustrating the timing of the CS and the US. Middle panel: representative O.O. EMG before learning. Lower panel: representative O.O. EMG after learning. (**d**, **e**) the CR% (**d**) and EMG response topographies (**e**) across two habituation and ten acquisition training sessions in ChR2/paired, ChR2/unpaired, and mCherry/paired groups ( = 8 rats each; ^*^ and ^#^ indicate significant differences between the ChR2/paired group and the ChR2/unpaired and mCherry/paired groups; ^*^or ^#^*P *< 0.05, ^**^ or ^##^*P *< 0.01, ^***^ or ^###^*P *< 0.001; two-way ANOVA with repeated measures followed by Tukey post-hoc test). Data are represented as mean ± s.e.m. The rat drawing was drawn by Guang-yan Wu according to the present experiment.

Next, we tested whether optogenetic activation of the right caudal mPFC pyramidal neurons is a sufficient CS for establishing DEC in rats. Rats were stereotactically injected with either pAAV 2/8-CaMKIIα-ChR2-mCherry or pAAV 2/8-CaMKIIα-mCherry and implanted with an optrode consisting of a fiber optic connula with two recording electrodes (insulated stainless steel wires, 76.2 μm inner diameter) directly attached to the optical fiber (200 μm core diameter, 0.39 numerical aperture) targeting the right caudal mPFC. Moreover, a guide cannula was implanted into the right caudal mPFC at an angle of 45° (Fig. 2a). One week after surgery, a part of the ChR2-treated (ChR2/paired group) and all of the mCherry-treated (mCherry/paired group) rats were conditioned by using a delay paradigm in which they received paired presentations of the optogenetic stimulation CS (470 nm, 350 ms, 10 mW/mm^2^, 20 Hz, 10 ms pulse duration) and periorbital shock US (Fig. 2b,c). Furthermore, another group of rats (ChR2/unpaired group) received random presentations of the same CS and US, which were explicitly unpaired in time. The rats of the three groups showed a very low frequency of spontaneous eyeblink (less than 7%) and did not differ significantly from each other during two habituation sessions (Fig. 2b,c). In contrast, there was a progressive increase in the percentage of CR (CR %), reaching asymptotic values, in the ChR2/paired group across acquisition sessions 1–10, which was due to associative learning, as the ChR2/unpaired and mCherry/paired group did not show increases in responding across acquisition sessions (Fig. 2d,e).

**Figure 3:**
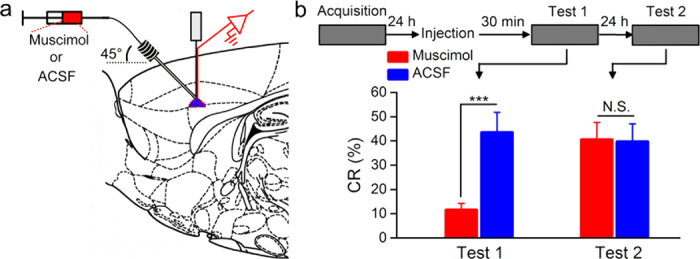
Muscimol injection prevents the expression of CR. (**a**) Injection of GABA_A_ receptor agonist muscimol (red) or ACSF (blue) into the right caudal mPFC. (**b**) Top: experimental scheme, Bottom: muscimol markedly impaired the CR expression compared with ACSF (left). However, this impairment was reversible when the same rats were retested 24 h later in the absence of muscimol (right; n = 9 rats for ACSF group and n = 10 rats for muscimol group; N.S., not significant, ^***^P < 0.001; two-tailed unpaired Student’s t-test). Data are represented as mean ± s.e.m.

To further confirm that the associative learning was due to the optogenetic activation of neurons in the right caudal mPFC as a CS paired with the US, we injected the conditioned rats with GABA_A_ receptor agonist (i.e., muscimol) to reversibly inactivate the right caudal mPFC before test (Fig. 3a,b). As expected, muscimol inactivation markedly impaired the expression of CR compared with ACSF (Fig. 3b, left). However, this impairment was reversible when the same rats were retested on the next day without injection (Fig. 3b, right). Together, these data suggest that direct optogenetic activation of the right caudal mPFC pyramidal neurons is a sufficient CS for establishing DEC in rats.

### Stable performance requires a period of time

Interestingly, although the above results suggest that optogenetic activation of the right caudal mPFC pyramidal neurons as a CS was sufficient for establishing DEC, the CR% after acquisition was relatively low (~40%) compared with results typically obtained with either a tone CS (~90%)^0^ or electrical stimulation of caudal mPFC as a CS (~90%)^7^. This phenomenon prompted us to perform a set of experiments to explore the possible reason. Other rats were injected with pAAV 2/8-CaMKIIα-ChR2-mCherry and underwent identical surgery, habituation, and acquisition training as the abovementioned ChR2/paired group (i.e., Mudium 10 + 40 group) except for the number of acquisition sessions or the parameter of optical stimulation. However, it can be observed that increasing the number of acquisition sessions (i.e., Long-term 10 + 40 group; 20 consecutive acquisition sessions; Supplementary Fig. S3a,b) did not produce a significant increase in appearance of the CR. Moreover, increasing the power density (i.e., Enhanced 10 + 40 group; 470 nm, 350 ms, 20 mW/mm^2^, 20 Hz, 10 ms pulse duration; Supplementary Fig. S3a,c) or pulse duration (i.e., 30 + 20 group; 470 nm, 350 ms, 10 mW/mm^2^, 20 Hz, 30 ms pulse duration; Supplementary Fig. S3a,d) of light failed to evoke similar levels of CR% compared with the CR% with a peripheral CS in our previous studies^0^. In addition, increasing the frequency of light pulse (i.e., 2 + 8 group; 470 nm, 350 ms, 10 mW/mm^2^, 100 Hz, 2 ms pulse duration; Supplementary Fig. S3a,e) did not increase the CR% yet.

Next, we calculated the CR% of the abovementioned groups in 10-trial blocks of the last acquisition session. Although the CR% of these groups on last acquisition session was different (Supplementary Fig. S5a), all group rats failed to successfully express CR during initial period of this session, and clearly showed progressive increases in CR% throughout the 10-trial block and reached a relatively high CR% (~80%) at last (Supplementary Fig. S4a). Moreover, the EMG response topographies of these groups for 10-trial block during the last acquisition session also indicated that there were increases in the CR amplitudes among all groups (Supplementary Fig. S4b–f). To quantify the deficits in CR performance during initial period, we directly calculated the number of trials and the latency to the criterion for stable performance of CR in these groups during the last acquisition session. Despite the number of trials and latency to criterion of each group were different, they all took a number of trials and latency to reach the criterion (Supplementary Fig. S5b,c). These data suggest that rats are able to acquire the memory of the CS–US association, but they could not stably express it during initial period of daily conditioning training even after the CR acquisition reached the asymptotic level.

**Figure 4:**
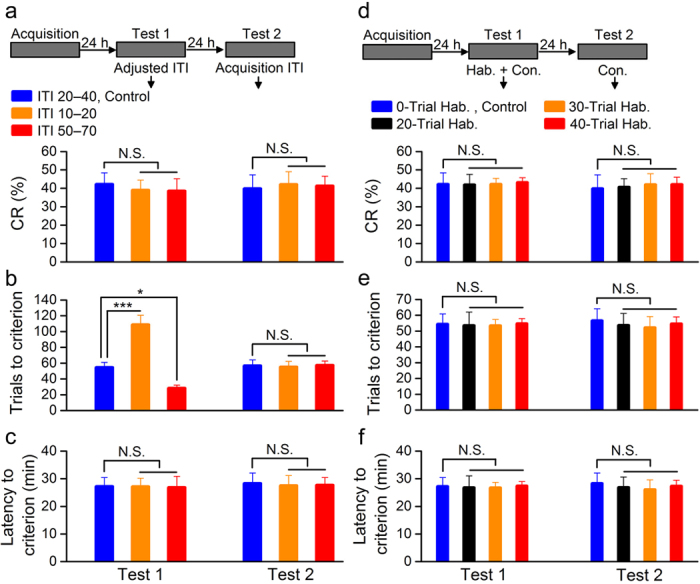
A period of time is required for stable performance of CR. (**a**–**c**) Top: training and testing scheme. Bottom: modification of intertrial interval (ITI) had significant effect on trials to criterion (**b**), but had no effect on CR% (**a**) or latency to criterion (**c**; n = 9 rats each; N.S., not significant, ^*^P < 0.05, ^***^P < 0.001; one-way ANOVA followed by Tukey post-hoc test). (**d**–**f**) Top: training and testing scheme. Bottom: modification of the proportion of habituation and conditioning training had no effect on CR% (**d**), trials to criterion (**e**), or latency to criterion (**f**; n = 9 rats for 0-trial Hab., control and 40-trial Hab. groups, n = 8 rats for 20-trial Hab. group, and n = 9 rats for 30-trial Hab. group; N.S., not significant, one-way ANOVA followed by Tukey post-hoc test). Data are represented as mean ± s.e.m.

To test the hypothesis that staying for a period of time in experimental environment rather than giving a number of paired presentations of the CS and US is critical for stable performance of CR, another three groups of rats underwent the same scheme as ChR2/paired group and tested 24 h later. When the frequency of paired presentations of the CS and US was modified [i.e., modifying intertrial interval (ITI)] and the total time of training was not changed, while the number of trials to the criterion for stable performance of CR was significantly higher or lower in ITI 10–20 and ITI 50–70 groups compared with that of ITI 20–40 group, there were no significant differences in the CR% or the latency to criterion among the three groups (Fig. 4a–c, left). Furthermore, the number of trials to the criterion did not differ significantly from each other when the same rats were retested on the next day without modifying the ITI (Fig. 4a–c, right). In addition, when the proportion of habituation and conditioning trial (the total of all trials was 100) was modified, there were no significant differences in the CR% or the number of trials and the latency to criterion among these groups (Fig. 4d–f, left). Moreover, they did not differ significantly from each other without habituation on the next day (Fig. 4d–f, right). Together, these results thus suggest quite strongly that rats fail to stably express CR during initial period of daily conditioning training after acquisition, and that the staying for a period of time in experimental environment rather than giving a number of paired presentations of the CS and US is a critical factor for reaching the criterion for stable performance of CR.

### The CS intensity and consistency affect the memory retrieval

**Figure 5:**
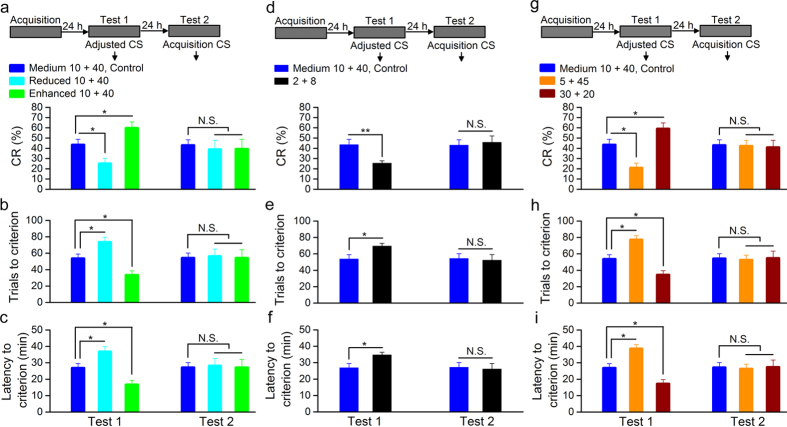
The CS intensity and consistency affect the memory retrieval. (**a**–**c**) Top: training and testing scheme. Bottom: decreasing the intensity of cue produced significant deficits in DEC retrieval, but increasing the intensity of cue produced significant improvements in CR performance (n = 8 rats for both reduced 10 + 40 and enhanced 10 + 40 groups and n = 9 rats for Medium 10 + 40, control group; N.S., not significant, ^*^P < 0.05; one-way ANOVA followed by Tukey post-hoc test). (**d**–**f**) Top: training and testing scheme. Bottom: changing the consistency of CS produced significant deficits in DEC retrieval (n = 8 rats for 2 + 8 group and n = 9 rats for Medium 10 + 40, control group; N.S., not significant, ^*^P < 0.05, ^**^P < 0.01; two-tailed unpaired Student’s t-test). (**g**–**i**) Top: training and testing scheme. Bottom: decreasing the intensity and consistency of cue produced significant deficits in DEC retrieval, whereas decreasing the consistency and increasing the intensity of cue produced significant improvements in CR performance (n = 8 rats for 5 + 45 group, n = 9 rats for Medium 10 + 40, control group, and n = 10 rats for 30 + 20 group; N.S., not significant, ^*^P < 0.05; one-way ANOVA followed by Tukey post-hoc test). Data are represented as mean ± s.e.m.

To examine the hypothesis that both the intensity and consistency of CS play critical roles in the retrieval process of the associative memory, other groups of rats underwent the same scheme as abovementioned ChR2/paired group and made retrieval test 24 h later. As expected, when optical CS with decreased or increased intensity (470 nm, 350 ms, 5 mW/mm^2^ or 20 mW/mm^2^ , 20 Hz, 10 ms pulse duration; i.e., changing CS intensity but keeping consistency) was used to retrieve the associative memory acquired with 10 + 40 CS, the rats with decreased intensity of CS (reduced 10 + 40 group) showed significant deficits in the associative memory retrieval, but the rats with increased intensity of CS (enhanced 10 + 40 group) displayed significant improvements in CR performance compared with rats with the same initial acquisition CS (medium 10 + 40, control group; Fig. 5a–c, left). However, there were no significant differences in the CR% and the number of trials and the latency to criterion among these groups on the next day when the same initial acquisition CS was used (Fig. 5a–c, right).

Interestingly, a 2 + 8 optical stimulation CS (470 nm, 350 ms, 10 mW/mm^2^, 100 Hz, 2 ms pulse duration; i.e., changing CS consistency but keeping intensity) used to retrieve the associative memory acquired with 10 + 40 CS, produced significant deficits in the associative memory retrieval compared with the initial acquisition CS (Fig. 5d–f, left). In contrast, no significant differences were observed between two groups on the next day with the same initial acquisition CS (Fig. 5d–f, right). Thus, these results were interpreted within the encoding specificity principle framework.

In addition, when a 5 + 45 or 30 + 20 optical CS (470 nm, 350 ms, 10 mW/mm^2^, 20 Hz, 5 or 30 ms pulse duration; i.e., changing both intensity and consistency of the CS) was used to retrieve the associative memory acquired with 10 + 40 CS, the rats of 5 + 45 group showed significant deficits in the retrieval of associative memory but the rats of 30 + 20 group displayed significant improvements in CR performance compared with these of the medium 10 + 40, control group (Fig. 5g–i, left). In contrast, there were no significant differences in the CR% or the number of trials and the latency to criterion among these groups on the next day when the same initial acquisition CS was used (Fig. 5g–i, right). Together, these data, thus, strongly support our hypothesis that both the intensity and consistency of CS play critical roles in the retrieval of associative memory.

## Discussion

Using targeted and reversible pharmacological and optogenetic approaches in awake, freely moving rats, we show that optogenetic activation of a subpopulation of pyramidal neurons in the right caudal mPFC as a CS paired with a peripheral US is sufficient to establish DEC. In contrast, unpaired presentations of the optical CS and US in ChR2/unpaired group did not obtain CR across 10 d of training, indicating that CRs observed in the ChR2/paired group were due to associative learning. Several key studies using optogenetic approaches have reported that optical activation of lateral amygdala pyramidal neurons as an US produced fear conditioning^8^, directly activating a subset of cells involved in the formation of fear memory was sufficient to induce the behavioral expression of the fear memory^2^, and sparse optical stimulation of barrel cortex drove a perceptual decisions and learning^9^. However, to our knowledge, we are the first to demonstrate that direct activation of pyramidal neurons in the right caudal mPFC as a CS is sufficient to acquire DEC in rats.

Although optical activation of the right caudal mPFC pyramidal neurons as a CS was sufficient to support DEC, the acquisition CR% was relatively low (~40%) compared with results obtained with a electrical stimulation of caudal mPFC as a CS (~90%)^7^. One possibility is that optical stimulation activated a small number of mPFC pyramidal neurons, while electrical stimulation did not. However, the c-Fos results showed that large numbers of mPFC pyramidal neurons were activated by optical stimulation (Supplementary Fig. S2a–d) and increasing the power density of light failed to produce similar levels of CR% compared with a electrical CS in our previous study^7^ (Supplementary Fig. S4a). Moreover, in freely moving rats, *in viv*o recordings demonstrated that the population spike (PS) evoked by the optical stimulation CS did not increase significantly in the amplitude after the rats reached the criterion of stable performance of CR on the tenth acquisition session (Supplementary Fig. S6a–c), indicating that the number of pyramidal neurons activated by optical stimulation may be similar between pre- and post-stable performance, and is sufficient to support the retrieval process of the CR. Therefore, this is not the essential reason for the lower levels of CR%. Furthermore, the low level of CR% may be explained by the fact that only one subpopulation of neurons (pyramidal neurons) have been activated, whereas other populations of cells may be required as well for the full expression of the DEC. In contrast, electrical stimulation of mPFC stimulated not only all pyramidal neurons but also the GABAergic neurons, glial cells, fibers of passage, and so on^0^. These cells and output or input fibers in the mPFC may be also important for the DEC^4^. It is also possible that electrical stimulation of mPFC as a CS was not “pure” due to the spread of electrical current. For example, the spread of electrical current may also stimulate the meninges, leading to a somatosensory CS, which has been shown to be an effective and sufficient CS for EBC^6^. Indeed, in these previous studies of electrical stimulation^9^, the current intensity of electrical stimulation for each animals was set before training by increasing the test current until a behavioral response was observed. The current was then turned down in 5-μA increments until there was no observable behavioral response. Typical behavioral responses observed from the test stimulation included head turns, orienting responses, and ear movements.

Although previous studies have provided convincing evidence that the intensity of CS plays a crucial role in the acquisition process of EBC^6^, the role of the CS intensity in the retrieval process of EBC remains to be determined. Our results suggest that the intensity of CS also plays an important role in the retrieval process of EBC. In addition, knowledge about the role of CS consistency in the retrieval process of EBC also remains sparse. The present results also demonstrated that the consistency of CS plays a critical role in the retrieval process of EBC. Although previous studies have shown that the intensity of retrieval cue plays an important role in the retrieval of such memories as spatial memory^1^ and fear memory^2^, and that the consistency of retrieval cue plays an critical role in the retrieval of episodic memory 5, this is, to our knowledge, the first demonstration that both the consistency and the intensity of CS play critical roles in the retrieval process of EBC.

Although it is clear from the present results that optogenetic activation of a subpopulation of pyramidal neurons in the right caudal mPFC is an effective and sufficient CS for establishing DEC, other factors affecting the acquisition and retrieval processes of the DEC remain to be explored. Using optical activation of a population of cells as a CS may be of importance in understanding the cellular mechanisms underlying associative learning. Moreover, our results about the role of CS intensity and consistency in the retrieval process of EBC may provide an insight into understanding the mechanisms underlying the retrieval process of memory.

## Methods

### Animals

Adult male Sprague Dawley (SD) rats, weighing 350–400 g (3–4 months) at the time of virus injection, were individually housed in standard stainless steel cages on a 12:12 light/dark cycle with free access to food and water ad libitum. Behavioural experiments were performed during the light cycle. The room temperature was maintained at 25 ± 1 °C. All animal procedures were approved by the Animal Care Committee of the Third Military Medical University and were performed in accordance with the principles outlined in the National Institutes of Health Guide for the Care and Use of Laboratory Animals.

### Virus injection

Rats were anaesthetized with a mixture of ketamine (100 mg/kg, i.p., Gutian, Fujian, China) and xylazine (9 mg/kg, i.p.; Sigma-Aldrich, St. Louis, MO, USA) and fixed in a stereotaxic apparatus (Model 940, David Kopf Instruments, Tujunga, California, USA). To label a subpopulation of pyramidal neurons in the right caudal mPFC, we used a pAAV 2/8-CaMKIIα-ChR2(H134R)-mCherry or pAAV 2/8-CaMKIIα-mCherry. Virus titres were 3.41 × 10^12^ genome copy (GC)/ml for pAAV 2/8-CaMKIIα-ChR2(H134R)-mCherry and 8.44 × 10^12^ GC/ml for pAAV 2/8-CaMKIIα-mCherry. Vectors were obtained from Addgene and packaged by Neuronbiotech (Shanghai, China). A small craniotomy was performed and the virus (1.0 μl) was delivered into the right caudal mPFC using a glass micropipette (tip diameter 10–20 μm) attached to a 5 μl Hamilton microsyringe (51189; Stoelting, Wood Dale, Illinois, USA) at the following coordinates: +1.9 mm anteroposterior (AP), 0.9 mm mediolateral (ML), and −2.3 mm dorsoventral (DV) (Fig. 1a,b). The injection rate (0.05 μl/min) was controlled by a stereotaxic microsyringe pump (53311; Stoelting, Wood Dale, Illinois, USA). After injection the needle was left in place for 5 additional minutes and then slowly withdrawn. Finally, the incision was closed with sutures.

### Immunohistochemistry

The virus-infected and fiber-implanted rats were injected with an overdose of 10% chloral hydrate (1000 mg/kg, i.p., Kelong, Chengdu, China) and perfused transcardially with physiological saline followed by cold 4% paraformaldehyde (PFA; prepared in 0.1 M of phosphate buffer, pH 7.4) 90 min after optical stimulation, which was 100 epochs of 350-ms light pulse trains (470 nm, 10 mW/mm^2^, 20 Hz, 10 ms pulse duration), separated by a variable interval of 20–40 s (with a mean of 30 s). The brains were removed from the skull and stored in 4% PFA at 4 °C for 24  h, then transferred to a 30% sucrose/4% PFA solution at 4 °C for 48  h. 30 μm-thick coronal sections were cut on a freezing microtome (CM3050 S, Leica, Germany) and collected in cold phosphate buffer saline (PBS, 0.01 M, pH 7.4). For immunostaining, each slice was placed in PBS-T (PBS + 0.2% Triton X-100) with 2% normal bovine serum for 1 h then incubated with primary antibody at 4 °C for 24 h (Mouse anti-CaMKIIα 1:100, C265, Sigma-Aldrich, St. Louis, MO, USA; Rabbit anti-GABA 1:1000, PC213L, Merck Millipore, Billerica, MA, USA; Rabbit anti-c-Fos 1:100, sc-52, Santa Cruz Biotechnology, Dallas, Texas, USA). Slices then underwent three wash steps for 10 min each in PBS-T, followed by 1 h incubation with secondary antibody (Goat anti-rabbit conjugated to FITC, 1:100, ZF-0311, ZSGB-BIO, Beijing, China; Goat anti-mouse conjugated to FITC, 1:100, ZF-0312, ZSGB-BIO, Beijing, China). Slices were then incubated for 5 min with Hoechst (1:1000, 861405, Sigma-Aldrich, St. Louis, MO, USA) and underwent three more wash steps of 10 min each in PBS-T, followed by mounting and coverslipping on microscope slides. Confocal fluorescence images were acquired on a Carl Zeiss LSM 780 scanning laser microscope (Germany) using a 40 × oil immersion objective. Slices were also imaged on an Olympus BX53F fluorescence microscope (Japanese) using a 2 × air objective.

### *In vivo* optrode recording

3–4 weeks after virus injection, rats were anaesthetized by 10% chloral hydrate (400 mg/kg, i.p., Kelong, Chengdu, China) and their heads were placed in a stereotaxic apparatus (Model 940, David Kopf Instruments, Tujunga, California, USA). An optrode consisting of a fiber optic connula with a multi-wire electrode tightly coupled with an optical fiber (200 μm core diameter, 0.39 numerical aperture, FT200EMT, Thorlabs, Newton, New Jersey, USA), with the tips of the electrodes extending approximately 400 μm beyond the tip of optical fiber was used for simultaneous optical stimulation and extracellular recordings. Electrodes were made of 16 individually insulated nichrome wires (17.78 μm inner diameter, 761000, A-M Systems, Sequim, WA, USA), attached to a 20 pin connector. The optrode was lowered to the right caudal mPFC (+1.9 mm AP, 0.9 mm ML, and −3.0 mm DV) using a hydraulic micromanipulator (PC-5N, Narishige, Tokyo, Japan) (Fig. 1f). The optical fiber was connected to a 470-nm LED (M470F1, Thorlabs, Newton, New Jersey, USA) controlled by a pulse stimulator (Master-9, A.M.P.I., Jerusalem, Israel). The power density of light emitted from the optrode was calibrated to 10 mW/mm^2^. After light-responsive cells were detected, a series of light stimuli were conducted: 100 epochs of 350-ms light pulse trains (470 nm, 10 mW/mm^2^, 20 Hz, 10 ms pulse duration), separated by a variable interval of 20–40 s (with a mean of 30 s). Electrophysiological signals were band-pass filtered (0.3–5 kHz) using an 16-channel microelectrode amplifier (model 3600, A-M Systems, Sequim, WA, USA) and acquired with a data acquisition system (Powerlab 16/35, ADInstuments, New South Wales, Australia) with a sampling rate of 20 kHz. Data were analyzed with NeuroExplorer 4 (MicroBrightField, Williston, VT, USA), a neurophysiological data analysis software.

### Surgery

All of the rats used for behaviour training were anaesthetized with a mixture of ketamine (100 mg/kg, i.p., Gutian, Fujian, China) and xylazine (9 mg/kg, i.p.; Sigma-Aldrich, St. Louis, MO, USA) and fixed in a stereotaxic apparatus (Model 940, David Kopf Instruments, Tujunga, California, USA) 3–4 weeks after virus injection. After onset of anesthesia, an optrode consisting of a fiber optic connula with two recording electrodes (insulated stainless steel wires, 76.2 μm inner diameter, 790900, A-M Systems, Sequim, WA, USA) directly attached to the optical fiber (200 μm core diameter, 0.39 numerical aperture, FT200EMT, Thorlabs, Newton, New Jersey, USA) was implanted in the right caudal mPFC. Furthermore, a guide cannula (No. 62001, RWD, Shenzhen, China; external diameter: 0.67 mm, internal diameter: 0.30 mm) was implanted into the right caudal mPFC at an angle of 45°. Since the tip of the infusion cannula (No. 62201, RWD, Shenzhen, China; external diameter: 0.20 mm, internal diameter: 0.10 mm) extended 0.5 mm beyond the tip of the guide cannula, the final infusion position and tips of the recording electrodes and optical fiber were at the same following stereotaxic coordinates: +1.9 mm AP, 0.9 mm ML, and −2.3 mm DV. Rats were additionally implanted with four electrodes, made of insulated stainless steel wires (76.2 μm inner diameter, 790900, A-M Systems, Sequim, WA, USA) in the upper eyelid of the left eye (Fig. 2a). One pair of electrodes for delivering the shock US was implanted subdermally caudal to the left eye. The second pair of electrodes was implanted into the ipsilateral orbicularis oculi muscle to record its differential electromyography (EMG) activity. The electrode tips were bent as a hook to facilitate a stable insertion in the upper eyelid (Fig. 2a). Moreover, a bare silver wire (0.1 mm in diameter) was connected to four stainless steel skull screws as a ground. The seven wires were connected to a 8 pin mini-strip connector. The mini-strip connector, optrode, and guide cannula were cemented to the skull with dental cement (Fig. 2b). After the surgery, the animals were allowed 1 week of recovery.

### Behavioral procedures

Prior to acquisition training, all rats underwent two initial 50 minute habituation sessions in a plastic box (35 × 25 × 20 cm), housed within a sound- and light-attenuating chamber (Fig. 2b). Each rat was presented with the same 100 trials used in acquisition training (see below), only in the absence of the CS or US—providing a baseline spontaneous blink rate, prior to the rat’s exposure to any conditioning stimuli.

The eyeblink conditioning was carried out using a delay paradigm. The CS was a 470-nm, 350-ms optical stimulation (20 Hz or 100 Hz; 5 mW/mm^2^, 10 mW/mm^2^, or 20 mW/mm^2^; 2 ms or 10 ms pulse duration) of pyramidal neurons in the right caudal mPFC, which was delivered from a 470-nm LED (M470F1, Thorlabs, Newton, New Jersey, USA) controlled by a pulse stimulator (Master-9, A.M.P.I., Jerusalem, Israel). The US was a 100-ms periorbital electrical shock (100 Hz, 1 ms pulse duration, square, cathodal pulse), delivered from a stimulus isolator (ISO-Flex, A.M.P.I., Jerusalem, Israel), controlled by a pulse stimulator (Master-9, A.M.P.I., Jerusalem, Israel). The intensity of the shock US was carefully calibrated to give the minimal current required to elicit a discrete eyeblink response (2–3 mA). The US intensity was set before the first acquisition session and was not changed during the rest of the experiment. During all of the CS–US paired trials, the CS terminated simultaneously with the US (Fig. 2c). The daily acquisition session (day) consisted of ten 10-trial blocks, each of which comprised nine CS-US paired trials and one CS-alone trial. The trials were separated by a variable intertrial interval of 20–40 s (with a mean of 30 s). The ChR2/unpaired group received the same CS and US, but the US was presented with a random interval between 1 and 10 s after the CS onset.

### Drug injection

The GABA_A_ receptor agonist muscimol (Sigma-Aldrich, St. Louis, MO, USA) was dissolved in artificial cerebrospinal fluid (ACSF) consisting of (in mM): 126 NaCl, 5 KCl, 1.25 NaH_2_PO_4_, 2 MgSO_4_, 26 NaHCO_3_, 2 CaCl_2_, and 10 glucose (pH 7.35–7.40). The conditioned rats were injected with 1.0 μl of muscimol (1.0 mM) or 1.0 μl of ACSF into the right caudal mPFC 30 min before the test training (Fig. 3a,b). Infusion procedures for each animal included removal of the internal stylet from the guide cannula, insertion of a stainless steel infusion cannula that extended 0.5 mm beyond the tip of the guide cannula, infusion of the solutions at a constant rate of 0.1 μl/min, removal of the infusion cannula 5 min after the cessation of infusion, and finally reinsertion of the internal stylet. The constant injection rate was maintained using a microsyringe pump (53222V, Stoelting, Wood Dale, Illinois, USA).

### Data analysis

EMG activity of the orbicularis oculi muscle and the LFP signals were band-pass filtered (0.1–1 kHz and 0.3–100 Hz, respectively) using an 16-channel differential amplifier (model 3500, A-M Systems, Sequim, WA, USA) and acquired with a data acquisition system (Powerlab 16/35, ADInstuments, New South Wales, Australia) with a sampling rate of 10 kHz.

EMG data were analyzed off-line for quantification of CRs with the help of a home-made programs. The collected EMG data were full-wave rectified and integrated with a 1-ms time constant. The integrated EMG activity was calculated to the standard score compared to the mean of the baseline activity for the 0–300 ms before the CS onset in each trial. The mean plus four times standard deviation (SD) of the standard EMG activity during the baseline period of each trial was defined as the trial threshold. If the standard EMG amplitude during baseline period exceeded the trial threshold and lasted >10 ms, the trial was regarded as a hyperactivity trial and excluded from further analysis. Moreover, A trial was considered to contain the CR if the standard EMG amplitudes exceeded the trial threshold and lasted >10 ms during the period of 50–250 ms after the CS onset. The percentage of CR (CR%) was defined as the ratio of the number of trials containing the CR to the total number of valid trials. Stable performance of CR was judged with a criterion of more than 60% occurrence of the CR in a successful CR trial and subsequent 9 consecutive trials or subsequent all trials (when the number of subsequent all trials less than 9). The Latency to criterion was defined as the interval from training onset to the time point when the first trial that met the criterion for stable performance of CR appeared. In addition, the LFP of trials before and after reaching criterion of stable performance of CR were averaged.

### Statistical analysis

All of the data were expressed as the mean ± standard error of the mean (s.e.m.). The statistical significance was determined by a two-tailed unpaired Student’s *t*-test, by an one-way analysis of variance (ANOVA) followed by Tukey post-hoc test, or by a two-way ANOVA with repeated measures followed by Tukey post-hoc test using the SPSS software for the Windows package (v. 18.0). A value of *P *< 0.05 was considered to be statistically significant.

## Author Contributions

G.-Y.W., G.-L.L. and J.-F.S. designed the study, interpreted results and wrote the paper. G.-Y.W., G.-L.L. and S.-L.L. set up and performed experiments. H.-M.Z., C.C. and H.F. performed and helped with data analysis. All authors discussed and commented on the manuscript.

## Additional Information

**How to cite this article**: Wu, G.-Y. *et al*. Optogenetic stimulation of mPFC pyramidal neurons as a conditioned stimulus supports associative learning in rats. *Sci. Rep.* **5**, 10065; doi: 10.1038/srep10065 (2015).

## Supplementary Material

Supplementary Information
