# Supplementary material for: Optogenetic stimulation of mPFC pyramidal neurons as a conditioned stimulus supports associative learning in rats
Source: Sci Rep. 2015 May 14;5:10065. doi: 10.1038/srep10065 (PMC4431347; doi:10.1038/srep10065)
Supplement: Supplementary Information [file srep10065-s1.doc]

**Supplementary Information**

**Optogenetic stimulation of mPFC pyramidal neurons as a conditioned stimulus supports** **associative learning in rats**

Guang-yan Wu^1^,^2,*^, Guo-long Liu^2,3,*^, Hui-min Zhang^1^, Chong Chen^2^, Shu-lei Liu^1^, Hua Feng^3^, Jian-feng Sui^1,3^

^1^Department of Physiology, College of Basic Medical Sciences, Third Military Medical University, Chongqing 400038, China, ^2^Experimental Center of Basic Medicine, College of Basic Medical Sciences, Third Military Medical University, Chongqing 400038, China, ^3^Department of Neurosurgery, Southwest Hospital, Third Military Medical University, Chongqing 400038, China.

^*^These authors contributed equally to this work.

Email: jfsui2003@163.com

**Inventory of Supplementary Information**

**Supplementary Figure**

Supplementary Figure S1

Supplementary Figure S2

Supplementary Figure S3

Supplementary Figure S4

Supplementary Figure S5

Supplementary Figure S6

**1. Supplementary Figure**

**
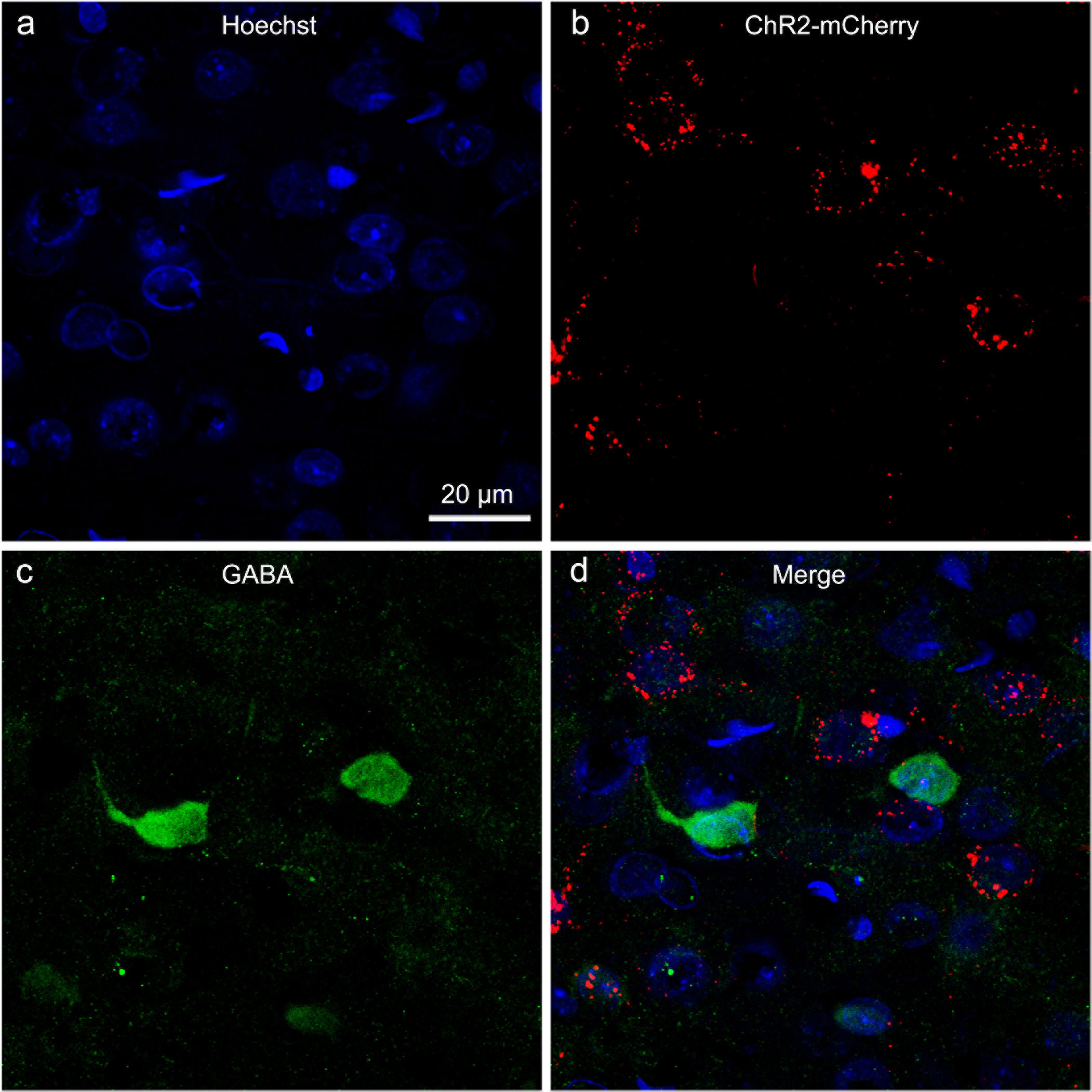
**

**Supplementary Figure S1 | GABA- and ChR2-mCherry-expressing cells do not overlap significantly.** (**a**–**d**) High-magnification view reveals membrane localization of ChR2-mCherry. No obvious overlap was detected between ChR2-mCherry-expressing neurons and GABA-expressing neurons.


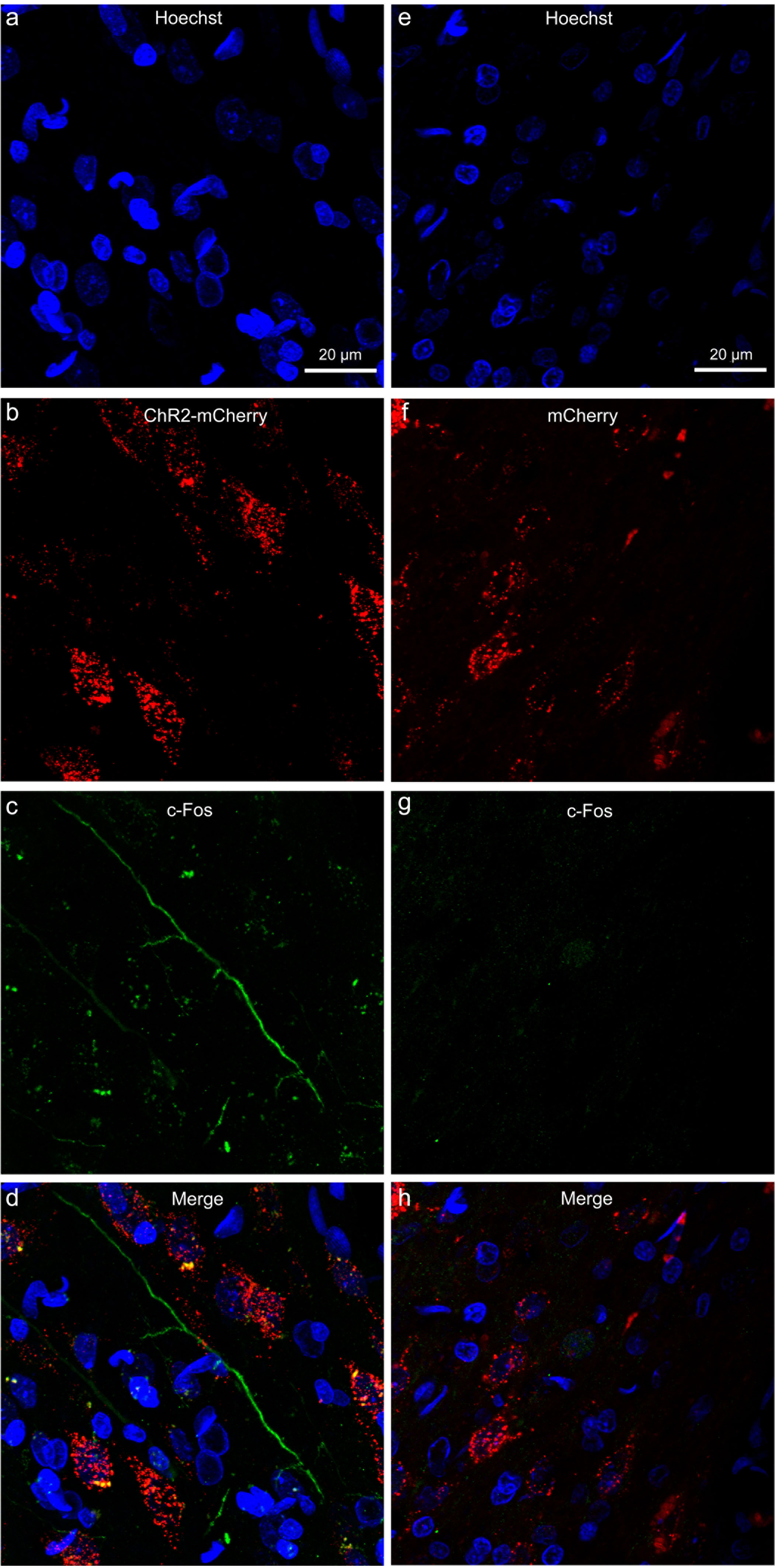


**Supplementary Figure S2 | Optogenetic stimulation induces c-Fos expression in cells expressing ChR2-mCherry but not mCherry.** (**a**–**d**) Representative cells in the right caudal mPFC after light stimulation in rats injected with pAAV 2/8-CaMKIIα-ChR2-mCherry. (E–H) Representative cells in the right caudal mPFC after light stimulation in rats injected with pAAV 2/8-CaMKIIα-ChR2-mCherry.

**
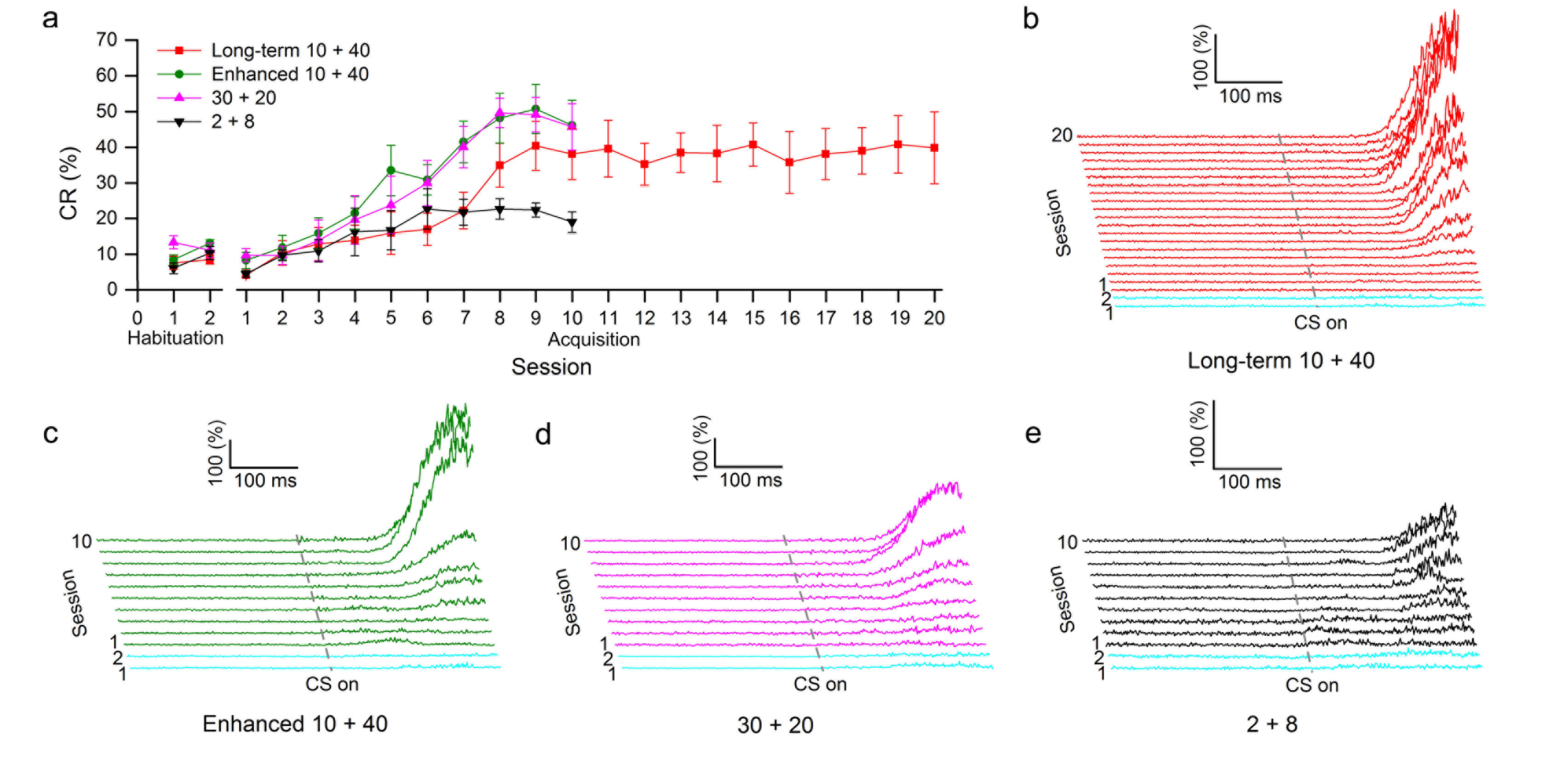
**

**Supplementary Figure S3 | Increasing the number of acquisition sessions, power density, pulse duration, or the frequency of light pulses do not evoke any significant increases in the CR%.** (**a**–**e**) the CR% (a) and EMG response topographies (**b**–**e**) across habituation and acquisition training sessions in long-term 10 + 40, enhanced 10 + 40, 30 + 20, and 2 +8 groups. (n = 7 rats for long-term 10 + 40 and 2 + 8 groups, n = 10 rats for 30 + 20 group, and n = 11 rats for enhanced 10 + 40 group). Data are represented as mean ± s.e.m.

**
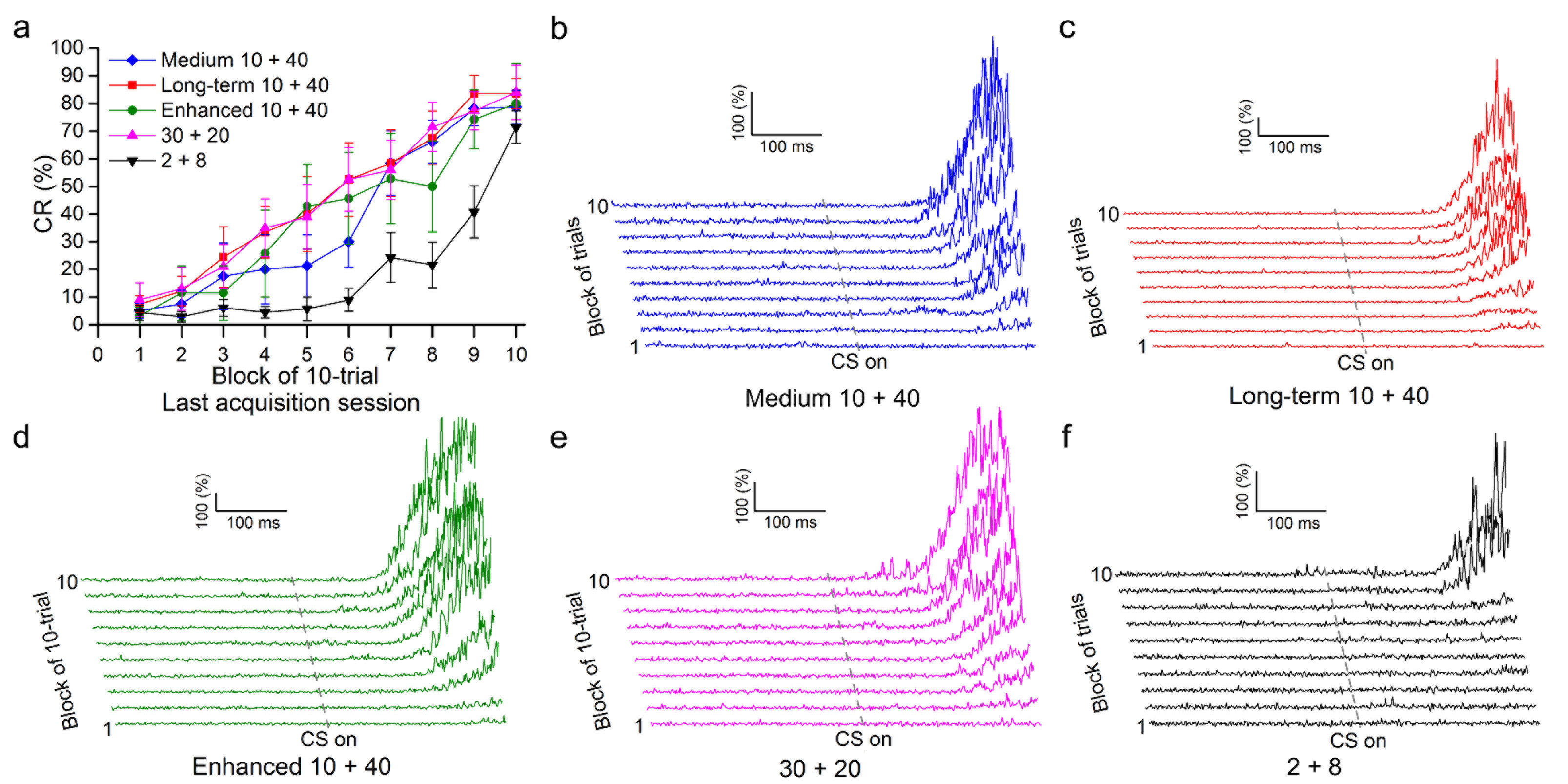
**

**Supplementary Figure S4 | Learned rats show progressive increases in CR% throughout the 10-trial blocks of the last acquisition session.** (**a**–**f**) the CR% (**a**) and EMG response topographies (**b**–**f**) across 10-trial blocks of the last acquisition session in medium 10 + 40, long-term 10 + 40, enhanced 10 + 40, 30 + 20, and 2 +8 groups (n = 7 rats for long-term 10 + 40 and 2 + 8 groups, n = 8 rats for medium 10 + 40, n = 10 rats for 30 + 20 group, and n = 11 rats for enhanced 10 + 40 group). A two way repeated measures ANOVA revealed that there was no significant interaction between groups and 10-trial blocks (F_(36,333)_ = 1.423; *P* = 0.060), but significant main effects of group (F_(4,37)_ = 3.298; *P* = 0.021) and of 10-trial blocks (F_(9,333)_ = 70.601; *P* < 0.001). Data are represented as mean ± s.e.m.

**
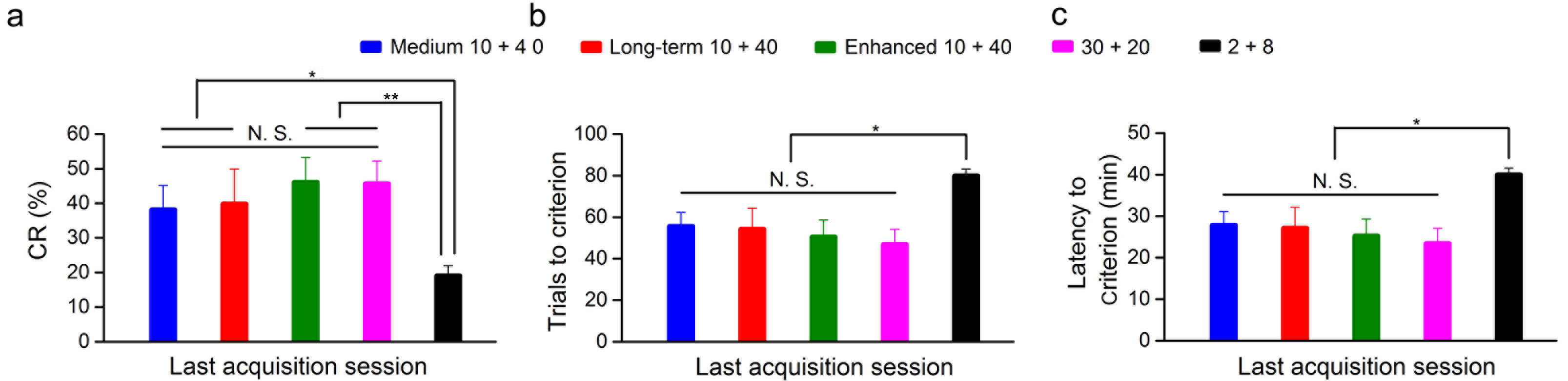
**

**Supplementary Figure S5 | Different performance of the associative memory acquired by distinct CSs on the last acquisition session.** (**a**–**c**) the CR% (**a**), trials to criterion (**b**), and latency to criterion (**c**) of the last acquisition session in medium 10 + 40, long-term 10 + 40, enhanced 10 + 40, 30 + 20, and 2 +8 groups (n = 7 rats for long-term 10 + 40 and 2 + 8 groups, n = 8 rats for medium 10 + 40, n = 10 rats for 30 + 20 group, and n = 11 rats for enhanced 10 + 40 group; N.S., not significant, ^*^*P* < 0.05, ^**^*P* < 0.01; one-way ANOVA followed by Tukey post-hoc test). Data are represented as mean ± s.e.m.

**
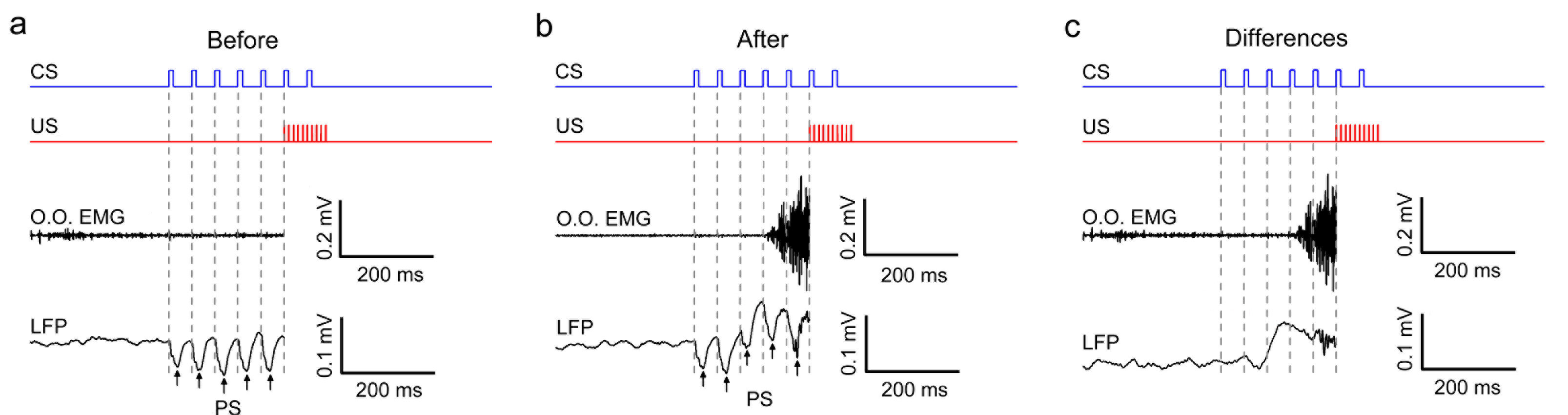
**

**Supplementary Figure S6 | The population spike (PS) evoked by the optical stimulation CS did not increase significantly in** **amplitude after the rats reaching criterion of stable performance of CR.** (**a–c**) Upper panel: the conditioning paradigm illustrating the timing of the CS and the US. Middle and lower panel: the averaged O.O. EMG and LFP of trials before (**a**) and after (**b**) reaching criterion of stable performance of CR, and of the differences (**c**) between (**a**) and (**b**). The data were collected from the tenth acquisition session of a animal in medium 10 + 40 group.
